# Music as aposematic signal: predator defense strategies in early human evolution

**DOI:** 10.3389/fpsyg.2023.1271854

**Published:** 2024-01-16

**Authors:** Joseph Jordania

**Affiliations:** University of Melbourne, Parkville, VIC, Australia

**Keywords:** human evolution and behavior, defense strategies, natural and sexual selection, aposematism crypsis, body painting, battle trance, cannibalism

## Abstract

The article draws attention to a neglected key element of human evolutionary history—the defense strategies of hominins and early humans against predators. Possible reasons for this neglect are discussed, and the historical development of this field is outlined. Many human morphological and behavioral characteristics–musicality, sense of rhythm, use of dissonances, entrainment, bipedalism, long head hair, long legs, strong body odor, armpit hair, traditions of body painting and cannibalism–are explained as predator avoidance tactics of an aposematic (warning display) defense strategy. The article argues that the origins of human musical faculties should be studied in the wider context of an early, multimodal human defense strategy from predators.

## Introduction

A valid defense strategy from predation is essential for the survival of any animal species. Consequently, articles and books dedicated to defense strategies in animal kingdom are plentiful (e.g., Ruxton et al., 2004; Caro and Girling, 2005; Gursky and Nekaris, 2007; Caro, 2009). At the same time, studies on the defense strategies of early humans have so far been strangely neglected. I probably need to clarify from the beginning, that works on the resistance of human organism against the viruses and other causes of inner pathologies are widely discussed in scholarly literature (particularly by virologists), but works about the defenses of early humans from their natural predators are notably absent. Two articles of Dutch ethologist Kortlandt (1965, 1980) represent rare exceptions. Only very recently, in June 2023, was a special interdisciplinary online conference “Defense Strategies in Early Human Evolution,” organized by the Jim Corbett International Research Center at Grigol Robakidze University in Tbilisi, Georgia (Jordania and Wade, 2023) with the participation of evolutionary biologists, paleoanthropologists, evolutionary psychologists, primatologists, neuroscientists, cognitivists, evolutionary musicologists, and conservationists, fittingly dedicated to the memory of Adriaan Kortlandt.

Let me first briefly outline my vision of the reasons for the strange neglect of this important topic in evolutionary scholarship. The first evidence for neglecting antipredator defenses in human evolution occurs in Charles Darwin’s book on human evolution (1871). When musing over the evolution on humans, Darwin abandoned his own greatest theoretical contribution to biological science–the theory of natural selection (Darwin, 1859). Instead, he proposed that an alternative theory, sexual selection, could better explain human evolution (Darwin, 1871). In his subsequent theory, tellingly, there was no place for natural predators of humans. According to Darwin, humans evolved in an environment lacking dangerous predators, an idea explicit in his book on human evolution by sexual selection:

The early progenitors of man were, no doubt, inferior in intellect, and probably in social disposition, to the lowest existing savages; but it is quite conceivable to consider that they might have existed, or even flourished, if, while they lost their brute-like powers, such as climbing trees, etc., they at the same time advanced in intellect. But granting that the progenitors of the man were far more helpless and defenseless than any existing savages, if they inhabited some warm continent or large island, such as Australia or New Guinea, or Borneo (the latter island being now tenanted by the orang), they would not have been exposed to any special danger. In an area as large as one of these islands, the competition between tribe and tribe would have been sufficient, under favorable conditions, to have raised man, through the survival of the fittest, combined with the inherited effects of habit, to his present high position in the organic world (Darwin, 1871, p. 173).

In the 1870s when Darwin wrote these words, there was no consensus about where humans had evolved, and all the major regions of the Old World (including Africa, Europe, and South-East Asia) represented potential candidates for “the cradle of humanity.” Today the scholarly community strongly agrees that humans evolved in Africa, which abounds in large predator species, including fierce competition among them. Therefore, Darwin’s theory of human evolution via sexual selection in a predator-free environment now seems unsustainable. Nevertheless, although no one remembers the Australia-New Guinea-Borneo “cradle of humanity” hypothesis, Darwin’s model of human evolution exclusively via sexual selection remains popular among many contemporary scholars, including evolutionary psychologists (e.g., Cronin, 1991; Miller, 2000; Richards, 2017).

A further discernible cause of the neglect of early human defense strategies emerged from the perspective of another great scholar of human evolution, Raymond Dart. Although initially Dart thought that hominins were small-time hunters and scavengers (Dart, 1925), he later underwent a complete change of mind and declared that early humans required no defense strategies because they were the apex predators and ruthless killers in their ecosystem (Dart, 1949, 1953). This model, known as the “killer ape hypothesis,” proposed in 1949, was later popularized by Ardrey (1961), and this image of our human ancestors as powerful big-game hunters had a commanding grip on the human psyche and still has an influential place in scholarship. The theory is particularly popular in explaining the apparently permanent human passion for warfare (e.g., Merker, 1984; Jones, 2008; Milam, 2019).

Critical reaction to the “killer ape hypothesis” came from two contrasting research paradigms. Authors of the first critical development, known as “man the hunted” hypothesis, argued that early humans were a weak prey species, whose best survival option was still to climb trees. This model was based on Brain (1981) diligent study of hominin taphological remains (*cf.* Hart and Sussman, 2005). On the one hand, as a positive development, this model acknowledged the immense pressure of predators on early humans, but on the negative side, it could not explain how such a weak primate prey species without any serious means of defense managed to live and sleep on the open savannah, much less travel outside of Africa, gradually becoming the widest distributed mammalian species on the planet.

The second development critical of the “killer ape hypothesis” argued that our ancestors were not big-game hunters, but rather scavengers. This model developed within the 1980s “new archeology” paradigm revolution (e.g., Binford, 1985; Bunn and Kroll, 1986; Blumenschine, 1986; Shipman, 1986; O’Connell et al., 1988a,b; Blumenschine and Cavallo, 1992; Dominguez-Rodrigo, 2002; Lupo and O’Connell, 2002; O’Bryan et al., 2019). When discussing the “scavenging hypothesis,” it is necessary to distinguish two very different modes of scavenging, which differ radically in terms of the defense/attack capabilities available to early humans: (1) passive scavenging, in which the carcass is accessed only after the original killer has left, and (2) confrontational (aggressive) scavenging, in which the original killer is chased from the carcass. Current consensus favors confrontational scavenging in early human evolution, but how early humans managed to chase the original hunter away remains a major question [e.g., “[M]icroscopic analyses indicate that cut marks on some bones overlay predators’ teeth marks, showing that the hominins arrived afterward. How they got meat away from scary scavengers is anyone’s guess.” Welker, 2017, p. 149]. The generally negative attitude toward scavenging in downplaying its possible role in the evolutionary past of our ancestors remains noteworthy: people prefer to see themselves as the descendants of big game hunters, not scavengers (e.g., Ehrenreich, 1997 on people’s overinflated attitude toward hunting and war).

A further possible reason for neglecting the defense strategies in humans’ evolutionary past might be the fact that it is very hard to distance ourselves from humanity’s current towering position in the contemporary world and objectively imagine the ancient past when our ancestors had to confront powerful predators in order to save their lives.

Hypothesis: Warning Display, or Aposematism, as a Defense Strategy.

The most recent early human defense strategy theory is aposematism. This new research paradigm naturally developed from the aggressive scavenging hypothesis, championed by “new archeology” (Binford, 1985; Shipman, 1986), a line of development furthered here. Considering the relatively obscure knowledge of this phenomenon, the following briefly outlines characteristics of aposematic defense.

Warning display (aposematism) is an important, but often neglected defense strategy in the animal kingdom. Unlike crypsis, which is based on the strategy of remaining invisible, silent, odorless, and fleeing as quickly as possible if discovered by a predator, aposematism is the alternative defense strategy of intimidating predators by remaining visible, being noisy, presenting odor, and, rather than fleeing when confronted by a predator, actively approaching and threatening the predator with body size, loud sounds, odors, and fearless behavior (Ruxton et al., 2004; Caro and Girling, 2005).

Species can be roughly divided into cryptic and aposematic categories, and these two different strategies fundamentally affect morphology and behavior. Cryptic species possess camouflaging body colors, try to stay put, move silently and generally remain silent, have no (or minimal) body odor, and flee as soon as they are discovered by a predator. Aposematic species, on the contrary, constantly try to be visible and noisy, have a stronger body odor, and if discovered by a predator, actively try to intimidate the potential predator with various body postures and colors, making aggressive sounds, emitting stronger body odor, and using threatening gestures and behaviors. As a result, aposematic species are more colorful, easier to see and hear, have more body odor, and do not run away when predators approach.

Aposematic signals serve two primary functions:

(1) *To intimidate* or to *warn* the predator by the display of size, colors, ornaments, noises, fearless behavior, and.
(2) *To educate*, or make their visual, olfactory, audible, and behavioral signals *remembered by the predator*.

Some of these signals are naturally weapons in themselves, such as a large body, antlers, big canines, venomous spike, or an aggressive behavior, referred to as *intrinsic aposematic signals* (Alonso and Jordania, 2023). Other aposematic signals, such as body odor or colors, or loud noise, which are not themselves harmful, can be categorized as *semantic aposematic signals*. Although both categories qualify as aposematic signals, the latter is the true meaning of aposematism.

Warning signals generally are much wider distributed in the animal kingdom than aposematism. Any animal species may display warning signals when cornered or frightened, or when they must face the attacker. A cornered rat against a cat or a cornered cat against a dog does not become an aposematic species although they display warning signals (they try to seem bigger, make loud sounds, display canines, and behave aggressively; e.g., Song et al., 2020). This is not an aposematic display, but a *startle*, or *deimatic* display (Rowe and Guilford, 1999). True aposematic animals do not display warning signals facultatively (sometimes, only when cornered), but constantly, such as skunks, porcupines, and venomous snakes (Ruxton et al., 2004; Caro and Girling, 2005; Caro, 2009).

Another very important feature of aposematic species is that all these audio, visual, olfactory, and behavioral signals just mentioned are, in fact, only bluff, the proverbial saber rattling, and they constitute merely the initial, primary, aposematic defense. In order to have a full, lasting, and sustainable aposematic defense, these animals must have some secondary, real defenses. So, in the case of a very hungry or uneducated predator still making an aggressive advance, aposematic animals need to hit the attacker with some kind of real weapon to inflict as much damage as possible. Aposematic secondary defense need not be fatal to the predator, but should be strong enough to be remembered as an unpleasant experience. Aposematic secondary defenses can involve various modalities, such as the venom of many snakes, spiders, and frogs, shock of the electric eel, unpalatable body of pufferfish, smelly spray of the skunk, or razor-sharp quills of the porcupine. Besides, aposematic animals can also simply escape predators by flying away (aposematic bird species) or by being overly aggressive (such as the honey badger), and of course, they can also employ the usual means of defense (teeth, antlers, body size) for secondary aposematic defenses.

## Aposematism and sexual selection via female choice

Aposematism and sexual selection via male competition have in fact many common features, and can act as complementary to each other: the features that are useful to scare away predators, are useful to scare away a rival as well. It is very different in sexual selection via female choice, where the features that attract female attention, are usually considered detrimental to the survival of males [as was implied by Darwin (1871), and was explicitly suggested by Zahavi in his “handicap principle.” See Zahavi (1975); but see Penn and Számadó (2020)]. It is important to remember that both sexual selection via female choice and aposematism utilize virtually the same signals: body colors, extra morphological additions to the body, loud vocalizations, and body odor. In both models it is crucial to impress the target audience (intraspecific females in sexual selection or extra-specific predators in aposematic display). Therefore, it is not accidental that aposematism was discovered by scholars in search of examples of sexual selection.

In 1867, while working on his book on sexual selection (1871), Darwin was struck by the colorful body of butterfly larvae. For Darwin only two kinds of colors were used in natural selection in the animal kingdom: defensive colors (camouflaging ones) and bright colors. Bright colors were automatically considered dangerous, and their existence was seen as justified only in the context of sexual selection. But Darwin could not explain why butterfly larvae, not yet sexually active, would advertise their bodies, rendering them easy for predators to see. Wallace, whom Darwin asked for help, explained the problem by proposing the mechanism of “warning flags” or “warning coloration.” Larvae that are unpalatable, explained Wallace, display warning flags to potential predators, so both the prey and predator species may escape harm. John Weir quickly organized experiments and proved Wallace’s idea right (Slotten, 2004, p. 263). Later Poulton (1890) came up with the term *aposematism* (“stay away sign” in Ancient Greek). Unfortunately, neither Darwin, Wallace, Weir, nor Poulton realized the true potential of the phenomenon of aposematism, which was at least a valid rival for the sexual selection theory, by explaining how bright colors could *help* some species survive by intimidating predators. So, for almost 150 years aposematism was considered a relatively rare phenomenon in the animal kingdom with hard-to-understand roots. Darwin was happy that Wallace helped explain the fact of colorful larvae without realizing that many cases of allegedly sexual selection could be cases of aposematic defense. Some recently published evolutionary encyclopedias still fail to include the term *aposematism* but have the term *warning coloration*, although color is only one of many modalities used by aposematic animals. At the same time, recent theorization indeed challenges (still a minority view I have to say) the sexual selection origins of such a widely known symbol of sexual selection, as the peacock’s train (Takahashi et al., 2008; Viegas, 2008; Jordania, 2011a, 2021; *cf.* Petrie et al., 1991; Petrie, 1994, 2021).

## Did humans use an aposematic strategy of defense?

Two principal suggestions have been proposed for humans being an aposematic species. In 1967 paleoanthropologist Louis Leakey proposed that humans are aposematic, in their being unpalatable to big cats, though he did not himself employ the term *aposematic*. Leakey used his personal experience to come to this conclusion, as he witnessed on more than one occasion an aversion to humans among lions. On one of his many lengthy field research visits to East Africa, lions entered the tent occupied by the scholar and his students (five lions on more than one occasion), and after sniffing human heads, left without attacking. Leakey believed that human smell somehow deterred lions:

I seriously believe that one of things which protected many early primates, including early man, in the defenseless days before he had weapons or tools, and when he was living on the ground, was that he was unpalatable to the carnivores.… Whether man’s natural immunity to large carnivores is smell by itself—they certainly sniff at us—or whether it is a combination of smell plus knowledge of how flesh tastes, I do not know, but I am convinced that a major defense mechanism of the earlier stages of protoman and early man was neither weapons nor canine teeth, nor claws nor physical strength, but his nature-endowed characteristic of being unpalatable, of not being good food for large carnivores (Leakey, 1967, p. 5).

While suggesting an aposematic interpretation, Leakey’s argument is weakened by the fact that human flesh is not itself unpalatable to historical and contemporary predators (see, for example, Corbett, 1944; Brain, 1981). Leakey’s interesting suggestion was recently reviewed by Paul Weldon from the Smithsonian Conservation Biology Institute, who proposed that humans are possibly chemically aposematic:

I propose that the body odor of humans and, historically, of hominins denotes chemical emitters that exhibit formidable defensive traits, including large body size, agility, vigilance, and the capabilities of deploying projectiles and other weapons and/or marshalling group defenses. This hypothesis maintains that selection acts against (1) offenders, including carnivores, that fail to avoid chemicals from hominins, and (2) hominins who fail to emit distinguishing chemicals, thereby give rise to a chemically mediated avoidance that is mutually beneficial, i.e., chemical aposematism (Weldon, 2018, p. 1).

It is widely known among behavioral ecologists, that aposematic strategy can by no means guarantee that the animal will be immune from predators, as predators are known to eat aposematic animals with very powerful secondary defenses (for example, unlucky skunks are eaten sometimes by very hungry dogs. See Ruxton et al., 2004). Similarly, humans can be still eaten by disabled predators (Corbett, 1944).

In books dedicated chiefly to this problem (Jordania, 2014, 2017), I argued that humans demonstrate *all the characteristics of aposematic features in every possible modality*: audio, visual, olfactory, and behavioral (not only in body odor, as pointed out by Leakey in 1967 and Weldon in 2018). The following several sections discuss the most important human aposematic characteristics. Some are relatively researched and known, but others will be presented for the first time in the context of human aposematism.

## Audio signals, singing in humans, or why do apes not sing?

The popularity of the idea that human choral singing grew from animal choruses used to defend territory is growing (Geissmann, 2000; Hagen and Bryant, 2003; Bannan, 2012; Jordania, 2014; Rice, 2014, p. 108; Harvey, 2017; Mehr et al., 2021; Savage et al., 2021; Leongómez et al., 2022; Nettl, 2022). Singing is a behavior overwhelmingly distributed in arboreal and aerial ecosystems (among the tree-living and flying species). Humans are among the very rare terrestrial species that sing (Jordania, 2020), though arguably some carnivores (e.g., wolves and coyotes) can also sing, and sing in choruses (Harrington, 1989; Hagen and Bryant, 2003; Hagen and Hammerstein, 2009). Despite some interesting parallels, the (adaptive) capacity for matching controlled synchrony of all four dimensions of sound production—pitch, duration, amplitude and timbre–provides a varied armory unmatched by any other species. The nature and the evolutionary reasons of the appearance of these abilities is still another little-discussed problem (for example, see Podlipniak, 2023). Here we need to remember, that another type of vocal signal, the *roar*, can be a very effective in deterring predators as well, particularly as startle signals (Raine et al., 2019; Kleisner et al., 2021). And although roaring is louder than singing and can communicate the strength better than any other audio signal (Raine et al., 2019; Kleisner et al., 2021), it requires much more energy, can damage the vocal chords if used excessively, and as a rule, is used only for the most critical situations (typically during the actual confrontation, as a *startle* signal, used by *both aposematic and non-aposematic animals*), not as a continuous vocal signal, such as singing, which can go on for hours by aposematic species (Turnbull, 1961; Pieslak, 2009; Knight and Lewis, 2017).

Early humans came down from the trees, and tree-living birds and primates (including a lesser ape, gibbons) are among the most ardent singers, so it would be logical to propose that our arboreal common (humans and apes) ancestor was a singer. The long-standing question that comes with this suggestion is why do terrestrial apes not sing? I propose that the question should be different—why did early humans not stop singing, as virtually all the arboreal species do when they visit the ground? Many singing and noisy arboreal species (birds and monkeys) maintain silence whenever they visit the ground as a cryptic defense strategy from potential ground predators (Catchpole and Slater, 1995; Jordania, 2020). Most likely, the ancestors of chimpanzees, gorillas and bonobos stopped singing for the same reason—maintaining cryptic cover while on the ground. In the case of non-singing arboreal orangutans, the most likely reason for them to stop singing was their *solitary lifestyle-* they do not even engage in grooming (Teboekhorst et al., 1990; Galdikas, 2005). On the other hand, in a strategically different move, early humans continued singing, therefore changing their survival strategy from cryptic into aposematic. I propose that not stopping singing was probably the first and deciding move toward the new aposematic strategy of defense in the hominin lineage, followed by the other elements of aposematic display (Jordania, 2011b, 2014, 2017). This fact is crucial for our understanding of the human tree-to-ground transition, and for understanding subsequent continuation of the two-media (song and language) underpinning universally of human culture that exists throughout the world.

Regarding the evolutionary origins of music, scholars today have virtually reached consensus that the evolutionary function of music (much like language) must be connected to the establishing of social connections among group members (Butcher, 1919; Blacking, 1973; Aiello and Dunbar, 1993; Dunbar, 1996, 2010; Bannan, 1999, 2012; Brown, 2000, 2003; Dissanayake, 2000; Benzon, 2001; Hagen and Bryant, 2003; Hauser and McDermott, 2003; McDermott and Hauser, 2005; Bispham, 2006; Cross, 2006; Fitch, 2006; Hagen and Hammerstein, 2009; Grauer, 2011; Hoeschele et al., 2015; Honing et al., 2015; Harvey, 2017; Savage, 2019; Mehr et al., 2021; Savage et al., 2021; Jan, 2022; Leongómez et al., 2022). Since the common human-ape ancestor was probably not only a capable individual singer but also sang in choruses, it is logical to suggest that the human tradition of choral singing started while they were still in an arboreal ecosystem. The next development of arboreal singing (and group singing) was greatly expanded with a new addition, that of a group unity, synchronicity (e.g., Bispham, 2006; Patel, 2008; Large and Gray, 2015; Brown, 2023). But before treating the importance of rhythmic synchronicity, it is important to discuss another feature of human and animal choruses, often neglected: the use of dissonances in human cultures and the animal kingdom.

Singing in choruses can be based on consonances (nice sounding, “sweet,” non-tense) or dissonances (rough sounding, tense) combination of sounds or intervals. Scholars have mostly concentrated on the use of nice sounding consonances (e.g., Tasuku et al., 2010; Crespo-Bojorque and Toro, 2016). I suggest in the context of the defense *paying special attention to dissonances*. Singing in dissonant intervals greatly contributes to the creation of a more robust sound. In the light of behavioral ecology, dissonant intervals are the most potent vocal signal for creating (1) the loudest possible sound, (2) the most attention-grabbing sound, and (3) the most effective “Beau Geste” sound (when a small group wants to make an audio impression of a much larger group; Wren, 1924; Harrington, 1989; Tripovich et al., 2008).

Another question arises: we know that Dissonances comprise the intervals where maximal frictions between the overtones create a more robust sound, but are dissonances physically louder than consonances? In my opinion, dissonances *sound* louder, for several psychological reasons: (1) dissonances grab our attentions faster than consonances; (2) I suggest that those cultures who sing in dissonances, generally sing louder that the “consonant cultures.” So, all this might indicate, that dissonances are not objectively louder, but they *seem* louder. This remains to be experimentally tested and confirmed or rejected.

We also need to clarify the notion of “minor” and “major” seconds. As ethnomusicologists know very well, when we deal with traditional cultures, assumptions regarding “minor” or “major” seconds may lack precision, as the second in most cases is between the major and minor seconds. German ethnomusicologists even use a special term “schwebungsdiaphonie” (roughly translated as “roughness, beat two-part singing”) which is the interval smaller than major second, and larger than minor second–this interval is used in the most isolated singing traditions, particularly in isolated mountain regions (Jordania, 2006, 2011a, 2015).

Thus, dissonant harmonies, particularly the sharpest dissonant intervals, seconds, should be historically most widespread when a group (potentially both animal and human) tries to warn/scare the opponent or a predator. However, although this function of music seems to me an original evolutionary factor, there is no reason to deny other kinds of musical sounds in human evolutionary history, including the sweet-sounding consonances and gentle humming for early humans (I discussed this dichotomy of musical functions in Jordania, 2009).

In biological scholarship this useful quality of dissonances was known earlier to animal experts. In works on wolves and coyotes, scholars paid attention to the specific dissonant coordination of the chorus participants that created a more effective Beau Geste defense (Harrington, 1989; Hagen and Bryant, 2003; Hagen and Hammerstein, 2009; Jordania, 2014, 2017). Singing in dissonant intervals occupies a unique place in human polyphonic singing cultures as well (Jordania, 2006, 2015). Singing in dissonant seconds is found in the most isolated cultures in the most geographically isolated regions of the world, namely in the Himalayas among Tibetans, in North Japan among the Ainus, in mountain tribes of Papua New Guinea, in Afghanistan among Nuristanis, and among the mountain minorities of North Vietnam, the Caucasus, Balkans, Baltic, Central Africa, the Andes, etc. (for a full review of these cultures, with notated musical examples, see Jordania, 2006, 2015). Furthermore, some cultures (e.g., Aremai and Aba Tibetans, and Latvians), who demonstrate arguably the most dissonant singing, also have free rhythm. This suggests that *singing in dissonances might be an earlier element in human cultures than the development of rhythmic synchrony* (Jordania and Wade, 2023). The fact that dissonances are used widely in animal choruses (at least by wolves and coyotes), and that rhythm is mostly absent among animal species and choruses, strengthens the argument.

The introduction of rhythmically united, synchronous singing was a revolutionary development in the choral singing of early humans (Bispham, 2006; Patel, 2008; Large and Gray, 2015). With synchronous choral singing, particularly together with dancing, the effectiveness of the audio intimidating/warning system skyrocketed. There are no animal species that do not run from the loud “wall of sound” created by a group of humans. The actual effectiveness of singing against big cats in India (man-eating tigers) was first noted by Corbett (1944), story of Chowgarh tigers. Corbett does not specify if the singing was in dissonances, we only know that it was a group singing. Generally, the effectiveness of singing as a “tool for intimidation” increases from singing alone, to singing with more persons; and also, from singing without harmony, to singing with harmony, and even more to singing with dissonant harmony. Basically, all kinds of singing are effective, but group singing in dissonant harmonies is the most effective. African Pygmies also use singing when going through the jungle, or, when they believe there is a danger of the attack from leopards at night (Turnbull, 1961, p. 58; Knight and Lewis, 2017).

Apart from the strong direct, external effect on predators and competitors, rhythmic synchrony introduced a probably even more powerful *internal* effect on groups of singing humans through the *entrainment*, with the help of dancing or merely walking together in time (e.g., McNeill, 1995). Synchronous singing and synchronous physical exercise lead to a potent phenomenon called *battle trance*, an altered state of consciousness that still needs serious research (Hedges, 2002; Junger, 2010; Jordania, 2011a; Wade, 2016; Kartomi, 2023). In this altered state, both men and women often experience (1) loss of fear (aphobia), (2) loss of pain (analgesia), (3) loss of memory (amnesia), (4) loss of ability to think rationally (irrationalism), (5) loss of the sense of individuality (deindividuation), while (6) gaining collective identity (when fighting as a group), and (7) gaining super-physical strength during the confrontation. In this state humans can behave extremely altruistically toward “own” group members, to the point of sacrificing their own lives, while behaving extremely aggressively toward “others,” to the point of senselessly killing noncombatants (Jordania, 2011a; Wade, 2016). This state can be triggered suddenly by an unexpected attack on a loved one (e.g., when a child, accompanied by a mother, is attacked by an aggressive dog or by a criminal), or deliberately through special training sessions in which the most effective and trusted way for the professional military is the long rhythmic drill sessions of the new recruits (McNeill, 1995; Ehrenreich, 1997).

Therefore, the audio aposematic signals of human ancestors included singing, more precisely loud choral singing in dissonances, rhythmically synchronized, and augmented with foot stomping, hand-clapping, and hitting stones together (Fitch, 2006; Jordania, 2014; Brown, 2023; Fitch and Zuberbühler, 2023). Singing (with occasional roaring or shouting) in a low voice was another potent intimidating signal for opponents, as human males have an unexpectedly low range (octave lower than females; Morris, 2008; Jordania, 2017; Bannan et al., in press). Audio signals were augmented by a visual display of threatening body movements (the New Zealand Māori haka tradition is a good example). Probably most importantly, this synchrony was the key factor putting participants of such primordial choruses into the euphoric state of battle trance, in which participants lose the sense of fear and pain, obtain a common collective identity, and are religiously dedicated to their common goals. Contemporary Western combatants still use rhythmically precisely synchronized singing and dancing to achieve this state (Hedges, 2002; Pieslak, 2009; Junger, 2010; Villarreal, 2010; Jordania, 2011a; Wade, 2016; Kartomi, 2023). Adding dance moves (initially as threat display movements), also in perfect synchrony, contributed an additional emotional power to the initial group singing, as the precise synchrony of a great number of individuals created the visual image of a single monstrously big creature impossible to confront.

## Human aposematic visual signals

Humans have evolved a variety of visual aposematic signals:

*Bipedal posture*: Hardly any human morphological or behavioral trait has received so much attention from every possible perspective as bipedalism (e.g., Hewes, 1961; Du Brul, 1962; Fifer, 1987; Eickhoff, 1988; Lovejoy, 1988; Latimer and Lovejoy, 1989; Hunt, 1996; Isbell and Young, 1996; Dunsworth et al., 2003; Carsten, 2010; Carrier, 2011; Kwang Hyun, 2015). Among many ideas was a suggestion that, since plenty of animal species use bipedal threat displays to look taller in order to intimidate antagonists, the effectiveness of a bipedal threat display could have led hominins gradually to adopt permanent bipedal posture. This is a relatively well-known hypothesis, initially expressed by Livingstone (1962), Wescott (1967), and later by proponents Jablonski and Chaplin (2004) and Jordania (2014), pp. 99-101.

*Long legs*: Humans have unusually long legs, one of the longest proportionally among the apes. With the obvious slow movement achieved with their long legs (Cartmill, 1983; Heinrich, 2002; Carsten, 2010), it is natural to propose that longer legs were gradually developed in order to be taller, since a higher body profile makes humans less vulnerable to predator attacks (e.g., Blake, 2023). All the major predators (including lions and tigers) display respect and clear aversion toward the human bipedal posture and human height.

*Long hair on top of a head*: Nina Jablonski suggested that it was evolutionarily advantageous for hominins to retain the hair on their heads in order to protect the scalp as they walked upright under the intense African sun (Jablonski, 2008). Desmond Morris suggested that overgrown head hair was used as a species-specific morphological sign for hominins, visible from afar (Morris, 2008). To better understand the evolutionary function of human head hair, two significant facts are important to note: (1) if left alone, untrimmed human head hair grows about 1.5 meters long. After this, individual hairs fall out and are replaced (Morris, 2008); (2) most likely the initial style of hominin head hair was a tightly coiled bush on top and around the hominin head, very much like the contemporary untrimmed “Afro” style that all peoples of African origin (including pygmies and bushmen) grow naturally. I suggest the unusually long hominin hair on top of the head had the same purpose as long legs and bipedal posture–simply to look taller. An untrimmed “Afro” must have added about 20 cm to body height, as it is several times as big as the diameter of a human head. A survey of the tall military helmets of Napoleonic hussars, or the colorful headdresses of warriors of different indigenous tribes, reflects the perennial drive to look taller among human warriors. Later humans substituted high military helmets for the Afro-style bushy hair to fulfill the same function to look taller and visually more impressive to potential opponents and predators (Jordania, 2011a, 2014).

*Body painting*: Another potent visual signal comes from the use of color. Humans naturally change the color of their faces and upper body when offended or angry (blushing), and usually they turn red–the most aposematic color (Harvey and Paxton, 1981; Crozier, 2010). Apart from this legacy of biological evolution, humans also have a legacy of early cultural evolution for employing more drastic colors via body painting.

The beginnings of body painting go much deeper than any rock painting and most likely originated at least with the oldest use of various pigments (Mithen, 2005; Roebroeks et al., 2012). No human culture is known to be totally free of body painting. For many tribes body painting is an important part of identity. Body painting in many traditional societies also signifies the status of a person or the moment of life they are experiencing; it also constitutes a very important part of initiation ceremonies in many parts of the world. Body painting was a significant ritual for men going into a hunting session or to war, even for achieving the coveted state of battle trance. Body painting is still widespread. Apart from permanent body painting, many temporary body paintings are in use. Using a lipstick or an eyeliner pencil is so widespread that hardly anyone would consider them in the same category as body painting. Hundreds of thousands of years before the estimated appearance of the first cave paintings, our ancestors were using coloring materials–such materials have been found at several archeological sites, although scholars have never found cave paintings of such an ancient age. The most likely explanation is that the first paintings were in fact done on human bodies.

Stone nodules containing mineral manganese dioxide, which has been scraped with stone tools, have been found at several Neanderthal sites… As the Neanderthals have left no traces of pigment on cave walls or artifacts, the most likely explanation is body painting (Mithen, 2005, p. 230).

As noted, striving to become more visually impressive became paramount to early humans for safety reasons, thus any physiological or behavioral changes that led hominins to acquire a more impressive look (e.g., bipedalism, long legs and long hair, blushing, or body painting) gave certain hominin groups better chances of survival by intimidating predators and competitors more effectively. This approach places *natural selection, not sexual selection* via *female choice*, as the driving force behind the tradition of body painting (Jordania, 2011b), but it is virtually impossible in this case to exclude the effect of sexual selection.

According to a 2012 article in the *Proceedings of the National Academy of Sciences of the United States of America*, the most popular and enduring coloring substance–red ochre–has been in use “minimally” for 200–250 kya (Roebroeks et al., 2012; *cf.* Bednarik, 1997). The users in this case were European Neanderthals, locked behind the ice sheets of Ice Age Europe. The use of painting substances among Neanderthals was doubted by scholars for decades, but growing evidence suggests that painting was widely used in isolated Europe much earlier than the appearance of anatomically modern Cro-Magnons:

Identification of the Maastricht-Belvédère finds as hematite pushes the use of red ochre by (early) Neanderthals back in time significantly, to minimally 200–250 kya (i.e., to the same time range as the early ochre use in the African record) (Roebroeks et al., 2012, p. 1889).

Indications suggest that even *Homo heidelbergensis*, a much earlier, taller, and muscular ancestor of the *Homo neanderthalensis* who lived in Europe 600–300 kya, also used red ochre for about 400,000 years. This evidence, although not universally accepted, comes from the Terra Amata site (Roebroeks et al., 2012).

Is it possible that our ancestors used other substances before red ochre–temporary substances they could easily obtain and use to paint themselves before they started using durable substances like red ochre (red) and manganese dioxide (black)? The idea that coloring faces and bodies started long before the use of durable materials is not only plausible, but virtually unavoidable. Such readily available coloring substances would be colorful berries, clay, even earth, and above all, blood. Blood most likely was the earliest coloring substance that humans used, putting the timelines of the origins of human arts much earlier (e.g., Bunney, 1990).

In summary, human visual aposematic signals included bipedal locomotion, long legs, long tightly coiled hair on top of the head, colors given by earlier biological evolutionary processes (blushing) and by later cultural evolution—the use of body painting. In addition, there were other powerful elements of visual display connected to dance and visual synchrony, such as the already mentioned New Zealand Māori “Haka” example (Gibson, 2011).

## Olfactory signals and other nighttime defenses

The evolutionary function of olfactory signals was very different from the function of visual and audio signals. Visual and audio signals work during the actual confrontation with predators and competitors by intimidating them with threatening images and impressive sounds, but olfactory signals mostly served as a reminder of the fighting abilities of hominins and early humans in the state of battle trance. Olfactory signaling was badly needed when humans were asleep on the ground, without their powerful visual and auditory defense modalities, such as Leakey’s close encounters with lions at night in the Serengeti, when body odor became their only defender (Leakey, 1967). Only after achieving relative safety on the ground at night could our ancestors be able to move away from the trees and start their intercontinental travels, so this ability warrants considerable attention.

First, human body odor is considered one of the strongest among animal species (Viegas, 2011). This smell is achieved by overactive sweat glands. The prevailing theory for humans’ immense number of sweat glands holds that humans overactive sweat glands enabled them to stay cool under the African Sun (Jablonski, 2008; Aldea et al., 2021). But sweat does not have to be smelly to cool the body, and human sweat is extremely smelly, even for a species with such a poor sense of smell as ourselves. Since apart from recent historical times, human ancestors did not shower or bathe for millions of years, the strength of hominin body odor becomes more overpowering. In this connection, I suggest that the patches of hair in armpits and groin were developed primarily for their hyper-effective smell-producing ability. Alternative suggestions for underarm hair (e.g., Kohl and Francoeur, 2002; Hofer et al., 2018; see also Wedekind, 2007) as a sexual attraction tool, or as a friction-reducing tool do not seem very convincing, as most humans now diligently try to get rid of body odor, particularly when meeting the opposite sex, and humans who shave their armpits (including sportsmen) never report any complications from injuries. I would predict, that aposematic species in general would have more smell-producing glands.

### Evening concerts

Kortlandt (1973) made a brilliant (and mostly neglected) suggestion that one way to secure nocturnal sleep was to organize loud evening “concerts” to scare away potential predators, citing the behavior of chimpanzee groups who sometimes produce loud concerts before they sleep, and also the behavior of African tribes living in the forests, who organize the same kind of loud evening displays. It is difficult to measure how long such concerts would have gone on: a perfect example is that when pygmies do not feel safe, they continue such concerts throughout the entire night (Turnbull, 1961, p. 58 Knight and Lewis, 2017). The long tradition of organizing most concerts in human societies in the evenings might be a legacy of the evolutionary strategy for nighttime security: we feel more secure after socializing with a group at a loud common display of unity.

### Eyespots as nighttime defense

Eyespots (“false eyes”) are clearly visible marks on the body of an animal that resemble the shape of an eye. It is a popular aposematic (and startle) visual signal. They are extremely effective against predation and attacks from behind because most potential predators seek a certain moment for their attack, when their prey is not looking at them. Many predators (including lions and tigers), when they see that the intended prey has noticed them approaching, lose interest.

Contemporary humans learned the benefits of eyespots. For example, from the safety precautions often found in Australian parks against swooping birds are these two points: “Draw a pair of eyes and attach to the back of your hat or bike helmet,” and “Wear sunglasses on the back of your head.” The same safety mechanisms work effectively against man-eating tigers, and cheap plastic masks worn on the back of the head became effective in deterring the man-eating tigers of the Sundarbans national park from attacking humans (Waltl, 2016).

According to tacit agreement, humans do not have natural eyespots, and neither do apes. Eyespots are characteristic of much more primitive animal species, such as butterflies and many other insects, some reptiles and fish, and some birds. However, eyespots are also present on one of the most evolutionarily advanced animal species–big cats (Leyhausen, 1960). Many big cats have eyespots on the back of their ears, and most important, since the big cats are humans’ most common natural predators, they are also very sensitive in noticing eyespots on others. Humans, on the other hand, are very bad at noticing eyespots, and some struggle to see the eyespots on big cats even when told about them.

To elaborate, big cats’ eyespots on the back of their ears are their defense from an attack from behind. These eyespots are also clearly seen from the frontal side when cats have their ears flat on their heads (Leyhausen, 1960). There is a possibility that, with this flattening of the ears on their head, cats show their eyespots to any antagonists in front of them. When viewing the face of an angry big cat with flattened ears, their false eyes (black eyespots on the back of their ears) are clearly displayed, and are bigger and spaced much wider that their real eyes. This display of bigger and widely set eyes may trick an antagonist into believing that the animal in front of them is bigger than it really is.

So far as I know, I was the first in the scholarly literature to propose that humans may have eyespots (Jordania, 2011a) but fail to notice them because (1) humans are generally bad at noticing eyespots; and, more characteristically, (2) because we only have them when the eyes are closed (during sleep). A human’s “sleeping” face features eyebrows, arched upwards, and the eyelashes, arched downwards, thus forming a pair of readily visible ovals, or eyespots. It is not easy for humans to notice the resemblance of human eyebrows and eyelashes to the eye because, not being by nature a predator species, we are generally bad at noticing eyespots. But eyespots on human faces were not designed by natural selection for other humans to notice: they were designed to be noticed by big African predators, particularly from the big cat family, and all the cats are particularly sensitive to recognizing eyespots.

I suggest that since hominins started sleeping on the open savannah, those individuals with longer and more arched eyebrows were less attacked by prowling big cats during sleep, since it seemed to predators that hominins were even in sleep looking at them. Generation after generation, individuals with longer and more arched eyebrows and long eyelashes survived. Of course, after humans stopped sleeping on the open savannah, the pressure to have nicely arched eyebrows and long eyelashes disappeared, but we still admire faces with clearly defined and arched eyebrows and long eyelashes (one more possible common point between the forces of natural and sexual selection).

According to the generally accepted view, the main function of the human eyebrow is to prevent moisture, mostly salty sweat and rain, from flowing into the eye. Morris (2008), discussing the possible function of the eyebrow in human evolution, criticized this suggestion as non-effective, and suggested that the primary function of the eyebrows was to signal changing moods (Morris, 2008; *cf.* Godinho et al., 2018). No doubt eyebrows are excellent communicators of mood, but I suggest their primary function evolutionarily was as a defense at night. At night eyebrows simply saved lives from predator attacks, which served as a big evolutionary pressure to develop and maintain them. At the same time, it is possible, even likely, that eyebrows had more than one evolutionary function.

Therefore, olfactory signals, designed for securing the nocturnal sleep of our ancestors, gradually enabled them to move far from trees and start long journeys. Human body odor is powerful, and the patches of hair in the armpits and groin were the means to create more powerful body odor. The appearance of eyebrows (and eyelashes) provided another defense mechanism, eyespots on a sleeping face. Therefore, the combination of the evening loud concert with communal singing and dancing before sleep, strong body odor spread with the wind (hungry prowling predators usually move upwind), and eyespots all created an effective multilayered nocturnal defense strategy (Jordania, 2014, 2017).

## Behavioral signals

An aposematic strategy of defense requires that audio, visual and olfactory signals are reinforced by behavioral signals. Aposematic species are set to follow several behavioral strategies. The most important characteristic is that aposematic animals should not run away when confronted by a predator. Instead, aposematic animals stand their ground and try to intimidate the potential predator with the display of audio, visual, olfactory, and behavioral signs.

### Freezing

“Do not run!” is first universal advice to everyone who suddenly finds themselves in dangerous proximity to a predator. Popular belief that it would take a lot of courage not to run away when seeing a deadly predator at close quarters is not correct. As a matter of fact, people in life-threatening situations usually freeze and cannot move, even if they want to. Although some still may try to run away in a state of panic, the more natural (and often life-saving) response, also instinctive, is to freeze.

The life-saving potential of the human freezing response is still unacknowledged by the scientific community, yet other versions of the freezing response are acknowledged, including such well-known facts as that some animals stand perfectly still so that predators will not see them, and some animals freeze or play dead when touched in hope that the predator will lose interest (Cannon, 1932; Jansen et al., 1995; Walker, 2013; Roelofs, 2017). So, these two types of freezing are acknowledged: one can be called “cryptic freezing,” aimed at remaining unnoticed by a predator, and the other can be called “catatonic” or “passive” freezing. Humans also often react to imminent danger by freezing, which is sometimes is seen as a serious disorder:

Of the various action disorders, cognitive paralysis leading to “freezing” behavior or catatonia in the face of danger is the most serious, as it prevents any survival response during the impact phase of the incident … Common speech describes such behavior in terms such as “struck dumb,” “petrified,” and “frozen stiff.” (Leach, 2004).

But of interest here, and what Corbett mentions in the final scene of the documental story “Robin,” is a very different type of freezing, neither cryptic nor catatonic (Corbett, 1944). In this documental story Corbett describes a reaction on a sudden attack of a leopard on him and his dog Robin, with the words:

“Our reactions to the sudden and quite unexpected danger that had confronted us were typical of how a canine and a human being act in an emergency, when the danger that threatens is heard, and not seen. In Robin’s case it had impelled him to seek safety in silent and rapid retreat; whereas in my case it had the effect of gluing my feet to the ground and making retreat rapid or otherwise impossible.” (Corbett, 1944, p. 40).

This, third version of freezing I call “aggressive freezing” with a very different message to the predator. Passive freezing sends the message to the predator, “I am yours, I am not running away, and I am not fighting back, so there is no need for violence.” But “aggressive freezing” sends a very different message: “I am not running away because I am not afraid of you. I am warning you that if you come closer, I will fight you, and you will regret your decision to attack.” This, I suggest, is “aposematic freezing” or “aggressive freezing.” Such freezing is an important part of the defense strategies of aposematic animals (skunks, hedgehogs, porcupines, venomous snakes), who famously do not run away at the approach of predators.

## Cannibalism as an early human defense strategy

An important addition to the behavioral characteristics of early humans is the widespread tradition of cannibalism in human history. William Arens rejected cannibalism as a gross lie and exaggeration, created by European colonizers (Arens, 1979), but this position became untenable in the light of increasing contemporary knowledge that cannibalism had been widely distributed throughout human history around the world as a ritual practice (White, 2001). In 2011 I suggested that cannibalism was a major element of early human defense strategy. Corbett (1944), in Author’s note was arguably the first who noticed that when human corpses were left unburied after major epidemics, predator attacks on humans increased drastically. Even the slave route across East Africa (with a high mortality involved) was connected to the appearance of the infamous Tsavo man-eaters at the end of the 19th century (Kerbis Peterhans and Gnoske, 2001; Waltl, 2016).

A fascinating but often overlooked fact of deep-seated human cannibalistic aspirations is the ubiquitous use of words describing cannibalistic behavior as the highest expression of love and affection. When we express excitement on seeing a cute puppy, kitten, or baby, we often declare we want to eat (or swallow) them. And as much as I have enquired of people from various cultures, I have found that such expressions, linking cannibalistic behavior with utmost love and affection, are virtually universal to all cultures and languages (no formal studies yet to confirm or reject this prediction). This accords with another fact: in some cultures where cannibalism was practiced, the act of consuming someone’s flesh was considered an expression of great respect and love for the deceased (Conklin, 2011; Jordania, 2022). Cannibalizing worshipped figures (both human and animal deities) for religious reasons is also widespread. The Christian Eucharist (Holy Communion), in which congregants symbolically consume the flesh and blood of Jesus Christ, is one such example.

However, some cultures have another, opposite reason for cannibalism: hatred and the desire to fully annihilate an enemy. In many traditional societies where cannibalism was practiced, both reasons were valid. People ate their slain enemy with a different feeling than eating their own, much-loved tribe member. This is the natural difference between endocannibalism and exocannibalism (Dole, 1962; Metcalf, 1987; Dorn and Tenenbaum, 1996; Vilaca, 2000). At the same time, from the view of cannibalism as a defense strategy from predators via “predator education,” both reasons are evolutionarily valid, as it is important not to leave any bodies available to predators after the battle, whether those of friends or foes. So, all the possible reasons–love for kin, hatred for the enemy, or a desire to acquire their strength by eating them–were beneficial to eliminate the available human bodies to predators. “There is no one satisfactory and all-inclusive explanation for cannibalism. Different peoples have practiced it for different reasons, and a group may practice cannibalism in one context and view it with horror in another” (Encyclopedia Britannica, Cannibalism). I suggest, that although cannibalism was used for various reasons in different regions throughout human history, this practice came from a single powerful evolutionary reason and was favored by the forces of natural selection. The reason was eliminating the presence of hominin and human dead bodies in the environment, so that predators did not have ready access to corpses–a very potent reason, and the only available way to eliminate human corpses, accessible to our hominin ancestors.

## Secondary defenses

Many secondary defenses used by aposematic animals, such as venom, stings, spikes, horns, and canine teeth, are not applicable to human ancestors. Apart from these obvious means, aposematic secondary defenses could be a big body, oversized antlers, or simply the overaggressive character of the species (like badgers or Norwegian lemmings that are not shy to attack even approaching humans; Anderson, 1976). Paul Weldon’s description of human secondary defenses is apt here: “large body size, agility, vigilance and the capabilities of deploying projectiles and other weapons and/or marshaling group defenses” (Weldon, 2018, p. 1). I fully agree with Weldon’s suggestion that the effective use of projectiles must have been the key factor of early human secondary defense strategy. The importance of the human ability to throw stones and other projectiles with a great force is widely known (e.g., Calvin, 1983; Fifer, 1987; Isaac, 1987), and it is rightfully acknowledged as the key evolutionary factor that formed the human body, particularly the male upper body (e.g., Longman et al., 2020). The only correction that I would like to make to this idea is to shift the initial aim of throwing from *hunting* to *defense* from predators.

A careful comparison between the *hunting throwing* and *defense throwing* strategies shows that defense throwing was for many reasons much more effective than hunting throwing for early humans:

- The distance is much closer in defense throwing. When an attacking animal (say, a lion) approaches, throwing the rock is a choice. The later the throw, the closer the target, the deadlier the mechanical result. When throwing for hunting, the target prey animal (say, an antelope) tries to stay clear of the hunter, and getting closer to the prey is not easy;
- It is much easier to aim accurately and hit a target in defense throwing, simply because the target is running toward the person. In hunting throwing, when the hunter is approaching the prey most likely from the back, the target might start running away. These two factors make hitting the target in hunting throwing much more challenging;
- Defense throwing is also more effective because it has a better chance of striking vulnerable parts of the body. When a target is approaching, the most likely place a thrown rock will hit is the head. In hunting throwing, when the prey is generally running away, the most likely place to strike is the hind quarters;
- The size and the weight of the thrown missiles can differ in defense and hunting throwing. Much larger rocks can be used in defense throwing, as the distance required to make an effective shot is much smaller, whereas in more distant hunting throwing, the best sized rock ideally should be less than 0.3 kilo;
- In defense throwing, when an attacking animal is coming close to the point of contact, a thrower can lift and hurl a much bigger single stone using both hands, greatly increasing the size and the weight of the missile. A close-range overhead throw of a much bigger rock increases the damaging force;
- In defense throwing, when a target is approaching, the speed of the running animal inadvertently augments that of the thrown rock, in the same way the collision of two oncoming cars is more forceful than a back-front collision. Similarly in hunting throwing where the target is usually running away, the impact of the thrown rock is less;
- The psychological factor is also important. A person would use their full bodily strength, and possibly even the hidden reserves of their “supernatural strength” in the moment when an attacking lion is running toward them. Hardly the same desperate supernatural force will come to their aid when trying to hit an antelope for dinner.

Therefore, I strongly suggest *shifting attention from hunting throwing to defense throwing*. Even today, defense among human armies is considered less costly than attack (Weisel, 2019). Early humans were most likely small-time hunters, but at the same time, they were the kings of scavengers, *apex scavengers*. On one hand, it was extremely difficult (and rare) for early humans to kill a decent sized prey for the whole group. At the same time, in the context of defense throwing, which would occur when early humans tried to chase away the prime hunters, they could obtain a more regular protein-rich diet from the specialized hunter animal species.

The throwing ability that initially started as a defense strategy against big predators in Africa was turned into an attacking strategy against the same predators (primarily lions), but this time in chasing away the predators from their kills. This was the major shift in the life strategy of early humans (Jordania, 2014, 2017; Somerville, 2019). Although early humans at first avoided lions, their natural predators, later, after finetuning their audio-visual-olfactory intimidating display (AVOID), they started attacking and chasing lions off after the kill was made. So, instead of avoiding lions, early humans started searching (and following) lions. Humans became active “vulture-searchers” in order to know about the scavenging opportunities on the open savannah. This must have been the final step away from the patches of trees to open terrain.

## Conclusion and implications

The evolution of human defense strategies started as soon as the human-chimpanzee common ancestor descended from the trees, initially, by adhering to an aposematic defense strategy, gradually developing a full set of aposematic signals in every modality:

*Audio signals*: Apart from singing in synchrony, using dissonant harmonies, clapping hands, and hitting stones, the message was enhanced by stomping, roaring, and yelling in low-range voice. Basically, human chorusing retains features shared with gibbon calls that have been lost by relatives genetically closer to humans (chimpanzees and gorillas lost singing probably because of relying on a cryptic defense system after they became terrestrial, and orangutans probably stopped singing because of their solitary lifestyle); Audio signals were possibly the first and most important aposematic signals, that gave general direction toward the appearance of other (visual, olfactory and behavioral) aposematic signals in human evolution;

*Visual signals*: Erect bipedal “threat display” became the permanent mode of locomotion; long legs and long tightly coiled head hair were developed; colors (natural color changes related to anger, and cultural use of color substances, first temporary, then durable) for body-painting were developed, plus threatening coordinated body movements (precursor of dance, primordial Haka).

*Olfactory signals*: Great number of sweat glands, resulting in the strength of body odor, with patches of hair in the underarms and groin to make the odor more effective, helped to educate predators, and particularly, ensure nocturnal sleep security in the open.

*Behavioral signals*: Going into battle trance, developing the freezing instinct in critical moments, not running from predators, slow and awkward movements, and ritual cannibalism to deny predators easy access to human corpses, were all designed as a part of effective predator education. After early humans developed effective defense strategies, they started gradually using their increased defense potential for aggressive scavenging sessions as well, becoming an apex scavenger of the African Savannah (Shipman, 1986; O’Bryan et al., 2019).

On open savannah humans started following lion prides, registering their kills via vulture watching, and attacking feasting lions at their recent kills. The stratigraphy and timelines of human and lion distribution over the world suggests that early humans were following lions (Barnett et al., 2006; Jordania, 2014; Willems and Van Schaik, 2017; Somerville, 2019). Most likely, Homo habilis was already well equipped with the aposematic signals and crude projectiles. Quite amazingly, the ancient tradition of stealing kills from hungry lions in East Africa by the Dorobo tribe was recently recorded by the BBC EARTH field team, available freely on YouTube (See “Grasslands: Stealing meat from the mouth of lions Human Planet”).

Even today, humans retain many features of aposematic animals, from individual behavior to the behavior of various human groups, and even nation states, where aposematic (warning, threatening) tactics play a major, sometimes a leading, role in international politics. The aposematic nature of humans is a powerful legacy of our evolutionary history, and its serious study might become one of the promising directions of research of evolutionary biology and evolutionary psychology.

## Data availability statement

The original contributions presented in the study are included in the article/supplementary material, further inquiries can be directed to the corresponding author.

## Author contributions

JJ: Writing – original draft, Conceptualization, Investigation.
